# Exclusive breastfeeding duration within a cohort of indigenous Terena living in the urban area of Campo Grande, Central-West Region, Brazil

**DOI:** 10.1590/0102-311XEN201922

**Published:** 2024-03-25

**Authors:** Deise Bresan, Maurício Soares Leite, Teresa Gontijo de Castro, Aline Alves Ferreira, Elenir Rose Jardim Cury

**Affiliations:** 1 Faculdade de Ciências Farmacêuticas, Alimentos e Nutrição, Universidade Federal de Mato Grosso do Sul, Campo Grande, Brasil.; 2 Departamento de Nutrição, Universidade Federal de Santa Catarina, Florianópolis, Brasil.; 3 School of Population Health, The University of Auckland, Auckland, New Zealand.; 4 Instituto de Nutrição Josué de Castro, Universidade Federal do Rio de Janeiro, Rio de Janeiro, Brasil.; 5 Programa de Pós-graduação em Saúde e Desenvolvimento na Região Centro-oeste, Universidade Federal do Mato Grosso do Sul, Campo Grande, Brasil.

**Keywords:** South American Indians, Child Health, Health Promotion, Infant Nutrition, Health of Indigenous Peoples, Índios Sul-Americanos, Saúde da Crianças, Promoção da Saúde, Nutrição do Lactante, Saúde de Populações Indígenas, Indios Sudamericanos, Salud Infantil, Promoción de la Salud, Nutrición del Lactante, Salud de Poblaciones Indígenas

## Abstract

In Brazil, current information about breastfeeding indicators among indigenous living in the urban areas is lacking. This article describes the duration of exclusive breastfeeding and its associations with mother and child characteristics in a cohort of Terena infants. The study enrolled infants born between June 2017 to July 2018 (n = 42) and living in villages of the urban area of Campo Grande, Mato Grosso do Sul State, Brazil. Information was collected in four time-points. Variables on maternal sociodemographics and on maternal and child health characteristics were collected, respectively, during the antenatal and the first-month interviews. Variables on breastfeeding practices and bottle use were collected during the first-, six- and 12-month interviews. Associations were examined using Wilcoxon, Kruskal-Wallis, Pearson’s chi-square, and Fisher’s exact tests. The prevalence of exclusive breastfeeding duration to the ages of three and six months were, respectively, 50% and 11.9%. Compared to infants never introduced to bottles during the first three months of life, those bottle-fed had lower median duration of exclusive breastfeeding (15 versus 150 days) and lower prevalence of exclusive breastfeeding duration to the age of three months (22.7% versus 80%). Most Terena infants fell short of meeting the international recommended duration of exclusive breastfeeding until six months of age and suggested the negative impact of bottle use in the duration of exclusive breastfeeding.

## Resumo

No Brasil, não há informações atuais sobre indicadores de aleitamento materno entre indígenas residentes em áreas urbanas. O objetivo deste estudo foi descrever a duração do aleitamento materno exclusivo e suas associações com características maternas e infantis em uma coorte de lactentes Terena. O estudo incluiu crianças nascidas de junho de 2017 a julho de 2018 (n = 42) e residentes em povoados da zona urbana de Campo Grande, Mato Grosso do Sul, Brasil. As informações foram coletadas em quatro momentos. Variáveis sociodemográficas maternas e características de saúde materno-infantil foram coletadas durante o pré-natal e o primeiro mês de entrevistas, respectivamente. As variáveis sobre práticas de amamentação e uso de mamadeira foram coletadas durante as entrevistas realizadas no primeiro mês, seis meses e 12 meses. As associações foram examinadas pelos testes de Wilcoxon, Kruskal-Wallis, qui-quadrado de Pearson e exato de Fisher. As prevalências de duração do aleitamento materno exclusivo até os três e seis meses de idade foram, respectivamente, de 50% e 11,9%. Em relação aos bebês que nunca foram introduzidos à mamadeira durante os três primeiros meses de vida, aqueles que usaram mamadeira tiveram menor duração média de amamentação exclusiva (15 *versus* 150 dias) e menor prevalência de duração de amamentação exclusiva até os três meses de idade (22,7% *versus* 80%). A maioria dos lactentes Terena não atingiu a duração recomendada internacionalmente para o aleitamento materno exclusivo até os seis meses, sugerindo um impacto negativo do uso da mamadeira na duração do aleitamento materno exclusivo.

## Resumen

En Brasil no existe información actual sobre los indicadores de lactancia materna entre los indígenas que viven en áreas urbanas. El objetivo de este estudio fue describir la duración de la lactancia materna exclusiva y sus asociaciones con las características maternas e infantiles en una cohorte de lactantes Terena. Este estudio incluyó a niños nacidos entre junio de 2017 y julio de 2018 (n = 42) y que vivían en aldeas del área urbana de Campo Grande, Mato Grosso do Sul, Brasil. La información se recopiló en cuatro momentos. Las variables sociodemográficas maternas y las características de salud materno-infantil durante la atención prenatal y el primer mes de entrevistas, respectivamente, se recogieron para este estudio. Las variables sobre prácticas de lactancia materna y alimentación con biberón fueron recolectadas de las entrevistas realizadas en el primer mes, seis meses y 12 meses. Las asociaciones pasaron por las pruebas de Wilcoxon, de Kruskal-Wallis, el chi-cuadrado de Pearson y la prueba exacta de Fisher. La prevalencia de duración de la lactancia materna exclusiva fue del 50% hasta los tres meses de edad y del 11,9% hasta los seis meses. En comparación con los bebés que no utilizaron biberón durante los primeros tres meses de vida, los que usaron biberón tuvieron una duración promedio más corta de lactancia materna exclusiva (15 versus 150 días) y una menor prevalencia de lactancia materna exclusiva hasta los tres meses de edad (22,7% versus 80%). La mayoría de los bebés Terena no alcanzaron la duración recomendada internacionalmente para la lactancia materna exclusiva hasta los seis meses, lo que sugiere un impacto negativo de la alimentación con biberón en la duración de la lactancia materna exclusiva.

## Introduction

Worldwide indigenous children are affected by high rates of undernutrition, infectious and parasitic diseases, and generally higher rates of infant morbidity and mortality, when compared with non-indigenous children ^1^ ^,^ ^2^ ^,^ ^3^. Childhood obesity has also been reported among several indigenous groups, with its prevalence sometimes exceeding the prevalence registered for non-indigenous children ^3^ ^,^ ^4^ ^,^ ^5^. Additionally to the historical colonization and exploitation that the indigenous peoples experienced over time, the precarious socioeconomic conditions they face make these groups particularly vulnerable to health inequities, with high prevalence of food insecurity ^1^ ^,^ ^3^ ^,^ ^6^ and the double burden of malnutrition ^1^ ^,^ ^3^ ^,^ ^6^ ^,^ ^7^ ^,^ ^8^.

A similar scenario has been reported among Brazilian indigenous peoples. Data from the only national health and nutrition survey designed to specifically address the country’s indigenous populations living in demarcated and settled territories ^9^ showed that more than a quarter (25.7%) of children under five years old were stunted ^10^, 80.2% of the infants had anemia ^11^, and 46.2% of the women in reproductive age had overweight/obesity ^12^. This survey was also the first to provide nationally aggregated data on breastfeeding practices of Brazilian indigenous peoples, showing that breastfeeding was initiated for 97.5% of infants and toddlers and that about a third of children (34%) between six and 23 months of age were exclusively breastfed until the age of six months ^13^.

The benefits of breastfeeding for children’s and women’s health are well established in the literature ^14^ ^,^ ^15^. Nutrition during the first years of life has the potential to lay the foundation for health throughout an individual’s life course ^16^ ^,^ ^17^. The World Health Organization (WHO) recommends exclusive breastfeeding until six months of age and continued breastfeeding until two years of age ^18^. Interventions designed to protect, promote, and support breastfeeding, including its exclusivity until six months of age and adequate complementary food in the first two years of life, are considered priority actions to impact the prevention and reduction of the double burden of malnutrition throughout of the course of life ^17^. Exclusive breastfeeding until age of six months can play an even more important role among socioeconomically disadvantaged populations, especially where infectious and parasitic diseases are still frequent causes of death and where access to health services are difficult. In these environments breastfeeding also constitutes an important protective factor against child morbidity and mortality ^19^ ^,^ ^20^.

In Brazil, indigenous groups were not included in national nutrition surveys (which are conducted since the decade of 1970), nor they are included in national monitoring/surveillance of breastfeeding indicators that takes place at the primary health care level. It means that the reported improvements in the duration of breastfeeding indicators in Brazil over the years refer only to non-indigenous children ^21^ ^,^ ^22^ ^,^ ^23^. In addition, Brazil lacks information on breastfeeding indicators for the different indigenous ethnicities. Studies on the theme are scarce, and are limited to specific indigenous communities, representing only a small fraction of the 305 ethnic groups officially recognized in the country ^24^ ^,^ ^25^ ^,^ ^26^ ^,^ ^27^. None of these studies included indigenous people living in urban areas of Brazil. Thus, although approximately 40% of the Brazilian indigenous contingent live in urban settings ^27^, there is no current information about breastfeeding indicators among these groups.

This study aims to describe the duration of exclusive breastfeeding and its associations with maternal and child characteristics within a cohort of Terena infants living in the city of Campo Grande, the capital of the State of Mato Grosso do Sul, in Brazil.

## Methods

### Study location and population

The Terena population represents one of the five most numerous indigenous ethnic groups in Brazil, with an estimated population of 28,845 individuals in 2010 ^27^. They live mainly in the state of Mato Grosso do Sul, in the country’s Central-West Region ^28^. About a third of the Terena population lives outside Indigenous Lands (located in in rural areas), representing the Brazilian Indigenous ethnicity with the largest number of individuals living in urban areas ^27^. All four Terena villages officially recognized by the Government in the State of Mato Grosso do Sul are located in the city of Campo Grande ^29^. Of the 5,657 people who self-declared indigenous living in the urban area of Campo Grande in 2010, 66% reported belonging to the Terena ethnicity ^30^. In 2013, 565 Terena families lived in the four Terena villages of Campo Grande ^31^.

The Terena people were predominantly farmers, and their first contact with non-indigenous people took place around the sixteenth century ^32^. Over the years, they lost an important part of their original lands ^33^, making farming, hunting, and fishing difficult ^34^. The Terena migration to urban centres began in the 1910s, mainly to the city of Campo Grande ^35^, where they settled in the peripheries of the city ^35^ ^,^ ^36^. From the 1990s onwards, low-income housing (“urban villages”) emerged in Campo Grande, which were mostly populated by indigenous peoples ^37^.

Most previous studies investigating the Terena’s health profile were limited to those living in Indigenous Lands, which are located in rural areas ^38^ ^,^ ^39^ ^,^ ^40^ and only one included those living in urban area ^41^. Among the Terena living in rural areas, studies reported high prevalence of stunting among children under five years old, ranging from 16% to 44.4% ^38^ ^,^ ^39^ ^,^ ^40^. Ribas et al. ^38^ also registered precarious prenatal care, where 84.5% of the pregnant women had less than the six recommended prenatal consultations during pregnancy and 51.7% started receiving prenatal care at the second or third trimester of pregnancy. In addition, these communities also face unfavorable socioeconomic conditions, including low income ^34^ ^,^ ^38^, precarious housing conditions and, lack of basic sanitation ^38^. A previous study conducted with the Terena infants living in the urban area of Campo Grande (same population of this study) found that the prevalence of low birthweight was 2.3% and that infants had, on average, lower birth weight if they were living in households with makeshift cesspools (compared with those living in households connected to the public sewage disposal system). Infants from mothers who were obese prior to the pregnancy were, on average, grams heavier at birth than those born from eutrophic mothers ^41^.

This study enrolled all Terena pregnant women living in the four urban villages of Campo Grande (Água Bonita, Darcy Ribeiro, Marçal de Souza, and Tarsila do Amaral). Eligibility was determined by an estimated delivery date between June 1st, 2017, and July 31st, 2018. The identification and recruitment of the pregnant women and their infants had the help of community leaders and was made by active visits to all households in the villages. A total of 51 eligible pregnant women and 52 babies were found. Three mothers refused participation and four dropped out or were lost during follow up. Additionally, in this study we excluded one mother of twin babies and one mother who had a premature baby from analyses, since these perinatal characteristics represent potential confounding variables for the outcomes examined. Thus, this study involved 82% (n = 42) of the infants initially eligible to be enrolled in the cohort. Within the cohort, most pregnant woman had antenatal care delivered by the public health system (97.6%) and most mothers had their babies delivered at hospitals (92.8%). The remaining mothers had their babies at the primary health care level, being transferred to hospitals later.

### Data collection waves

Information on the Terena infants and their mothers was collected during four home visits: one during pregnancy and three during the first 12 months of life of the infants (first-, sixth-, and 12th-month interviews). Mothers answered structured questionnaires which were based and adapted from the *First National Survey of Indigenous People’s Health and Nutrition* (referred from here as the National Indigenous Survey) ^9^.

**Figure 1:**
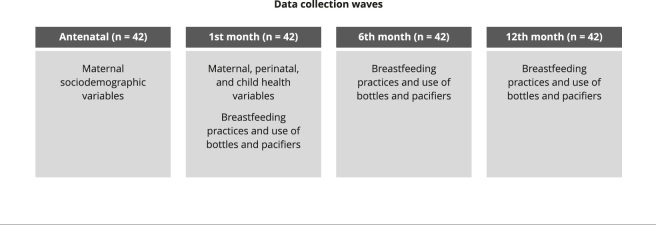
Cohort’s data collection waves and variables collected among Terena infants. Campo Grande, Mato Grosso do Sul State, Brazil (2017-2019).

Information on maternal sociodemographic characteristics were collected antenatally. Data on maternal and child health characteristics were obtained during the first-month interview. Variables on breastfeeding practices and bottle use were collected during the first-, sixth-, and 12th-month interviews (Figure 1).

### Maternal and child characteristics

The maternal sociodemographic variables collected were number of completed years of formal education, occupation, age, parity, partnership status and whether the mothers lived with their extended families (mothers or mothers-in-law), monthly per capita income (converted to USD in this article). The following information on maternal health an child perinatal characteristics were collected: trimester of initiation of prenatal care, number of prenatal care consultations and maternal pregestational weight (all obtained from health records held by the mothers - the pregnant women booklet of the Brazilian Ministry of Health), and child’s sex, birthweight, and type of delivery (all obtained from the infants’ health records - the Child Health Pregnant booklet of the Brazilian Ministry of Health).

Maternal height was measured at the first-month interview according to protocol described by Lohman et al. ^42^ and using a portable stadiometer (Alturexata, Belo Horizonte, Brazil) with accuracy of 0.1cm. Maternal pregestational body mass index (BMI) was calculated dividing the self-gestational pregestational weight (in kilograms) by the square of the height (in meters) and it was classified according to the WHO cut-off points ^43^.

### Variables on breastfeeding practices

This study used the definitions of breastfeeding and exclusive breastfeeding proposed by the WHO ^44^ and adopted by the Brazilian Ministry of Health ^45^. Therefore, breastfeeding was defined as “*when the child receives breast milk (directly from the breast or expressed), regardless of whether or not she receives other foods*” ^45^ (p. 13). The exclusive breastfeeding was defined as “*when the child receives only breast milk, directly from the breast or expressed, or human milk from another source, without other liquids or solids, with the exception of drops or syrups containing vitamins, oral rehydration salts, mineral supplements or medicines*” ^45^ (p. 13). All mothers, at all interviews, answered to questions inquiring about the infant breastfeeding practices and use of bottles and pacifiers, as described next.

Information on breastfeeding initiation was obtained from the following question, asked at the first-month interview: “Have you ever breastfed or are you breastfeeding the baby? (yes/no)”. Mothers were also asked if their infants were breastfed within the first hour of life.

Information on infants’ use of pacifier was obtained at the first-, sixth-, and 12th-month-interviews. At the first interview mothers were asked: “Has the baby used a pacifier since he/she was born? (yes/no)”. At the sixth- and 12th-month interviews, the mothers were asked the question: “Is <CHILD> using a pacifier? (yes/no)”.

Duration of exclusive breastfeeding was estimated by the following questions, asked to mothers at the first-month interview: “Since your baby was born, has he/she received water? tea? baby bottle with milk? what kind of milk? another type of liquid or food?” “If so, after how many days of life he/she had when received this liquid/food?”. Infants were considered in exclusive breastfeeding at that point if the answer to all the questions was “no”, or when they only received breast milk from a bottle. For mothers who answered “yes” to at least one of the listed liquids/foods, infants were considered no longer exclusively breastfed and exclusive breastfeeding duration was defined as the age at which the first food/liquid was introduced. For infants who were in exclusive breastfeeding at the first-month interview, at the sixth-month interview we asked their mothers: “Has <CHILD> started to eat/drink cow’s milk, goat’s milk, powdered milk, or other non-dairy milk human? tea? juice? water? fruits, vegetables, greens? soup? beans? porridge? eggs? beef, pork, chicken, fish? rice, flour, bread, pasta? any other food?” “If so, how many days of life he/she had when he/she received this liquid/food?”. Infants were considered in exclusive breastfeeding at that point if the answer to all the questions was “no”. For mothers who answered “yes” to at least one of the listed liquids/foods, infants were considered as no longer being exclusively breastfed and exclusive breastfeeding duration was defined as the age at which the first food/liquid was introduced. Finally, for infants who were in exclusive breastfeeding at the six-month interview, at the 12th-month interview we repeated the same question asked at the sixth-month interview to estimate the age at which EBF was discontinued for this group.

Information on any breastfeeding duration was collected at the 12th-month interview, by asking the mothers: “Is <CHILD> breastfeeding or was she/he breastfed?”. When the answer was “yes, she/he is still breastfeeding”, it was considered that the child continued being breastfed until the age of 12 months or beyond. For mothers who answered “yes, she/he was breastfed before”, it was considered that the child was no longer breastfed at 12 months of age.

### Data analysis

We used descriptive statistics to report relative frequencies, proportions, minimum and maximum values, and medians (interquartile ranges). Wilcoxon rank-sum and Kruskal-Wallis tests were used to compare the median duration of exclusive breastfeeding within, respectively, dichotomic and polytomous covariates. Comparisons of proportions of babies exclusively breastfed until the age of three months within the categories of sociodemographic variables were made using Pearson’s chi-square tests and Fisher’s exact tests. This last test was performed when counts of participants in any cell were inferior to five. Analyses were performed using the Stata 16.0 software (https://www.stata.com). All p-values were two-tailed (adopting 5% as the level of significance).

### Ethical aspects

This study was conducted according to the guidelines laid down in the Declaration of Helsinki and all procedures involving human subjects were approved by the Human Research Ethics Committee of the Federal University of Mato Grosso do Sul (1992298 and 2252520) and by the National Commission for Ethics in Research of the Brazilian Ministry of Health (2051925 and 2295467). The study was approved by all community leaders and informed consent was obtained from all participant mothers.

## Results

At the prenatal period, approximately one quarter of the infants’ mothers (23.8%) were working outside home and 45.2% had a maximum of eight years of formal education completed. Most mothers (60%) had pregestational excess weight and 38.1% were primiparous. About half of the mothers started prenatal care after the 16th week of pregnancy and 52.4% had less than six prenatal consultations during pregnancy. Among the infants, 52.4% were males and 38.1% were born by caesarean section (Table 1).

**Table 1:** Sociodemographic and health characteristics of the Terena mothers and infants. Campo Grande, Mato Grosso do Sul State, Brazil, (2017-2019).

Characteristics	n	%
Mothers		
Years of formal education completed		
5-8	19	45.2
9-11	23	54.8
Occupation		
Works at home	32	76.2
Works outside home	10	23.8
Age (years)		
< 21	16	38.1
21-27	14	33.3
> 27	12	28.6
Partnership status		
No partner	8	19.0
Partner	34	81.0
Living with own mother or mother-in-law		
No	26	61.9
Yes	16	38.1
Monthly per capita income [tercile (USD)]		
1st (43.00-68.99)	13	33.4
2nd (79.00-124.99)	13	33.3
3rd (125.00-308.00)	13	33.3
Parity (number of children)		
1	16	38.2
2	13	30.9
≥ 3	13	30.9
Pregestational BMI (kg/m^2^)		
Normal (18.5-24.9)	16	40.0
Overweight (25.0-29.9)	13	32.5
Obesity (≥ 30.0)	11	27.5
Prenatal care consultations		
< 6	22	52.4
≥ 6	20	47.6
Beginning of prenatal care		
By the 16th week of pregnancy	24	57.1
After the 16th week of pregnancy	18	42.9
Type of delivery		
Vaginal	26	61.9
Cesarean	16	38.1
Infants		
Sex		
Male	22	52.4
Female	20	47.6
Breastfeeding in the first hour of life		
No	13	30.9
Yes	29	69.1
Use of pacifier in the first month of life		
No	34	81.0
Yes	8	19.0
Bottle use in the first 3 months of life		
No	20	47.6
Yes	22	52.4

BMI: body mass index.

Note: N = 42. Missing data (n): monthly per capita income (3); pregestational maternal BMI (2).

The mean age (± SD, standard deviation) of infants’ mothers was 23.1 ± 5.6 years and their average monthly per capita income was USD 111.71 ± 67.59 (with minimum income of USD 43.00 and maximum of USD 308.00). Infants’ mean weight at birth (± SD) was 3,382 ± 442 grams. At the sixth- and 12th-month interviews, respectively 76.2% and 92.9% of the infants were being bottle-fed. These figures were 11.9% and 9.5%, respectively, for the use of pacifiers at the sixth- and at the 12th-month interviews. Approximately one in five infants were using pacifiers in the first month of life.

**Figure 2:**
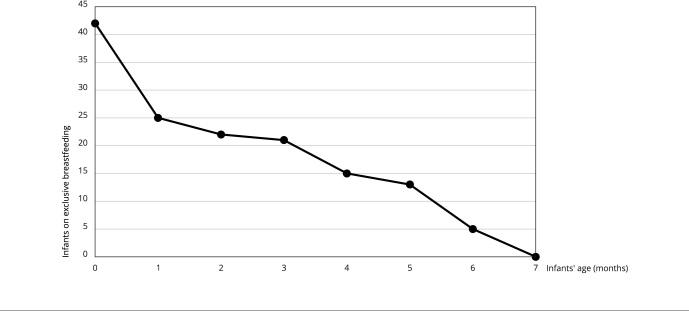
Prevalence of exclusive breastfeeding during the first seven months of life among Terena infants. Campo Grande, Mato Grosso do Sul State, Brazil (2017-2019).

All infants had breastfeeding initiated, approximately one third were not breastfed in the first hour of life and 85.7% were still being breastfed at the 12th-month interview. The median duration of exclusive breastfeeding (interquartile range) was 75 days (10; 150). Exclusive breastfeeding duration ranged between 1 to 180 days among the Terena infants. The prevalence of exclusive breastfeeding duration until the first month of life was of 59.5%, dropping to 50% until the age of three months and to 11.9% until six months (Figure 2).

Duration of exclusive breastfeeding and use of bottles in the first three months were significantly associated (Table 2). Infants who used bottles in the first three months of life had lower median duration of exclusive breastfeeding (15 days) when compared with those who did not (150 days). The prevalence of exclusive breastfeeding until the age of three months was almost four times lower among infants who were bottle-fed in the first three months of life (22.7%) when compared with those who were not (80%). The median duration of exclusive breastfeeding and the proportion of infants exclusively breastfed until the age of three months showed no statistically significant associations with the other maternal and child variables examined (Table 2).

**Table 2:** Median duration (interquartile range) and prevalence of exclusive breastfeeding until the age of three months according to sociodemographic and health characteristics of the Terena mothers and infants. Campo Grande, Mato Grosso do Sul, Brazil, 2017-2019

Characteristics	n (N = 42)	Median exclusive breastfeeding, days (interquartile range *)	Prevalence of exclusive breastfeeding until the age of three months (%)
Mothers			
Years of formal education completed		p = 0.898 **	p = 0.757 ***
5-8	19	60.0 (10.0-150.0)	47.4
9-11	23	90.0 (10.0-150.0)	52.2
Occupation		p = 0.413 **	p = 1.000 ^#^
Works at home	32	75.0 (12.5-150.0)	50.0
Works outside home	10	52.5 (5.0-150.0)	50.0
Age (years)		p = 0.052 ^#^	p = 0.155 ***
< 21	16	90.0 (45.0-165.0)	68.7
21-27	14	17.5 (4.0-120.0)	35.7
> 27	12	20.5 (7.5-150.0)	41.7
Partnership status		p = 0.686 **	p = 1.000 ^##^
No partner	8	60.0 (15.0-150.0)	50.0
Partner	34	75.0 (10.0-150.0)	50.0
Living with own mother or mother-in-law		p = 0.334 **	p = 0.525 ^#^
No	26	30.0 (10.0-150.0)	46.1
Yes	16	90.0 (15.0-165.0)	56.2
Monthly per capita income [tercile (USD)]		p = 0.505 ^#^	p = 0.115 ***
1st (43.00-68.99)	13	90.0 (90.0-150.0)	76.9
2nd (79.00-124.99)	13	20.0 (10.0-150.0)	46.1
3rd (125.00-308.00)	13	30.0 (5.0-120.0)	38.4
Parity (number of children)		p = 0.686 ^#^	p = 0.926 ***
1	16	75.0 (15.0-150.0)	50.0
2	13	90.0 (10.0-150.0)	53.8
≥ 3	13	20.0 (10.0-120.0)	46.1
Pregestational BMI (kg/m^2^)		p = 0.791 ^#^	p = 0.853 ***
Normal (18.5-24.9)	16	90 (9.0-150.0)	56.2
Overweight (25.0-29.9)	13	90 (15.0-120.0)	53.8
Obesity (≥ 30.0)	11	15.0 (11.0-150.0)	45.4
Prenatal care consultations		p = 0.542 **	p = 0.537 ***
< 6	22	45.0 (5.0-150.0)	45.4
≥ 6	20	90.0 (15.0-150.0)	55.0
Beginning of prenatal care		p = 0.229 **	p = 0.212 ***
By the 16th week of pregnancy	24	22.5 (9.0-135.0)	41.7
After the 16th week of pregnancy	18	90.0 (20.0-150.0)	61.1
Type of delivery		p = 0.334 **	p = 0.204 ***
Vaginal	26	25.0 (10.0-150.0)	42.3
Cesarean	16	90.0 (22.5-150.0)	62.5
Infants			
Sex		p = 0.694 **	p = 0.537 ***
Male	22	30.0 (11.0-120.0)	45.4
Female	20	90.0 (9.0-150.0)	55.0
Breastfeeding in the first hour of life		p = 0.901 **	p = 0.739 ***
No	13	90.0 (30.0-90.0)	53.8
Yes	29	30.0 (10.0-150.0)	48.3
Pacifiers use in the first 3 months of life		p = 0.834 **	p = 1.000 ***
No	34	75.0 (10.0-150.0)	50.0
Yes	8	52.5 (11.5-120.0)	50.0
Bottle use in the first 3 months of life		p < 0.001 **	p < 0.001 ***
No	20	150.0 (105.0-165.0)	80.0
Yes	22	15.0 (5.0-60.0)	22.7

BMI: body mass index.

Note: missing data (n): monthly per capita income (3); pregestational maternal BMI (2).

* Upper limit of the 2nd quartile and lower limit of the 3rd quartile;

** Wilcoxon rank-sum test;

*** Pearson’s chi-square test;

^#^ Kruskal-Wallis test;

## Fisher’s exact test.

## Discussion

This study described the duration of exclusive breastfeeding and its associations with maternal and child characteristics within a cohort of Terena infants living in the city of Campo Grande. All Terena infants included in the study had breastfeeding initiated. At three months of age, half infants were no longer receiving breastfeeding exclusively and at six months of age only one in ten infants were in exclusive breastfeeding. The median duration of exclusive breastfeeding was shorter and the prevalence of exclusive breastfeeding until the age of three months was lower among infants who were bottle-fed within the three months of life, compared with those who were not.

The prevalence of exclusive breastfeeding until the age of six months among the Terena infants (11.9%) fell far shorter than the prevalence registered by the National Indigenous Survey (34% nationally and 28.5% among the children living in the Central-West Region of Brazil) ^13^. When compared with studies conducted with specific Brazilian indigenous communities, the prevalence of exclusive breastfeeding until the age of six months registered among the Terena was lower than the prevalence registered among the Katukina, Nukini, Nawa, and Poyanawa in the state of Acre (35%) ^26^ and higher than the median duration registered among the Xakriabá in the State of Minas Gerais (7.27 days) ^25^. However, the comparison between the Terena breastfeeding data (collected longitudinally) with data collected by the National Indigenous Survey ^9^ and specific studies ^25^ ^,^ ^26^ (collected cross-sectionally) is hampered by different methodologies used to estimate exclusive breastfeeding duration and by these referred studies involving only rural communities ^9^ ^,^ ^25^ ^,^ ^26^.

A more favorable scenario was observed regarding the duration of any breastfeeding for 12 months or more among the Terena (85.7%), which was similar to rates registered for other indigenous peoples in Brazil ^24^ ^,^ ^26^ and higher than rate registered for non-indigenous Brazilian children - 53.1% in 2019 ^46^.

Compared with non-indigenous people, worse breastfeeding indicators have been described among indigenous peoples from Australia ^47^, Canada ^48^, the United States ^49^, Mexico ^50^, and New Zealand ^51^. Findings from other parts of the world have reported significant changes in breastfeeding patterns among indigenous populations. Among native peoples in Mexico, the prevalence of exclusive breastfeeding until six months of age decreased from 46% to 34.5% between 1999 and 2006 ^50^. Among Aborigines in Australia, Brown et al. ^52^ and Cromie et al. ^53^ also reported that women living in remote areas were more likely to exclusively breastfeed for longer compared with those who were living in urban centres. These results suggest that changes in traditional breastfeeding practices are associated with urbanization and introduction of modern Western practices such as the use of infant formula milks ^53^ ^,^ ^54^. The most recent urbanization processes experienced by the indigenous peoples in Brazil ^55^ ^,^ ^56^, the increased exposure to Western values and behaviors, which include standards of beauty and female behaviors, and the use of substitutes for breastmilk, bottles, and pacifiers ^19^, may have potentially intensified changes in breastfeeding and complementary feeding practices among these peoples ^19^. In addition, when indigenous groups settle in urban contexts, they tend to belong to the lower socioeconomic strata, resulting in lower access to social and labour rights and protection, including support and protection for breastfeeding ^57^. In Brazil, the effects of the recent labour legislation reform on breastfeeding practices in the medium and long term need to be monitored, since they may result in less access to labour rights ^58^.

Decisions about breastfeeding initiation and duration also encompass a conjunction of other factors, which range from sociocultural and economic aspects and the quality of health services to the individual aspects such as family support, maternal work and labour legislations, smoking, obesity, depression, age, and maternal education ^57^. In our study, exclusive breastfeeding duration and prevalence of exclusive breastfeeding duration until three months of age showed no significant associations with the maternal factors investigated. This may be due to the small numbers of infants included in the analyses, which may have compromised the statistical power for the comparisons performed. Our study indicated, however, that the use of baby bottles was significantly associated with lower duration of exclusive breastfeeding/prevalence of exclusive breastfeeding duration until the age of three months, corroborating findings registered for non-indigenous children in Brazil ^59^ ^,^ ^60^ ^,^ ^61^. The negative health and nutritional consequences of baby bottle use are widely known and described in the literature ^62^. As described in this study, 52.4% of the Terena infants were being bottle-fed at three months of age, 76.2% at six months, and 92.9% at 12 months. The prevalence of bottle-feeding at six months of age among the Terena is concerning, since it was almost three times higher than the proportion registered in the National Indigenous Survey in 2008/2009 (29.9%) ^13^ and approximately 20% higher than the prevalence registered for non-indigenous two-year-olds (51.2%) in 2019 ^23^.The use of baby bottles, the lower exclusive breastfeeing duration registered in this study, and the high frequency of surgical deliveries registered among the Terena in previous study ^41^ represent components that reflect the medicalization of childbirth and childcare practices in these communities ^19^ ^,^ ^63^ ^,^ ^64^. Also note that in Brazil, barring some exceptions, the health care available for indigenous living in urban area is the system designed for non-indigenous populations. Thus, these groups do not have access to the Indigenous Health Care Subsystem, linked to the national public health system. This subsystem provides differentiated health care that is sensitive to the cultural and epidemiological specificities of the indigenous context ^65^.

Over the decades, Brazil has successfully implemented a set of strategies that reversed the low frequencies of breastfeeding recorded in the 1970s. These included, among other actions, changes in the labour laws that granted maternity leave for four months and allowed women to be absent from work to breastfeed; new rules for the marketing of food and products for infants; the creation of human milk banks; the implementation of the Baby-Friendly Hospital Initiative, with protocols that favor breastfeeding; the training of health professionals; and national breastfeeding campaigns ^21^ ^,^ ^66^ ^,^ ^67^. Thus, the unfavorable indicators of exclusive breastfeeding duration and bottle-feeding registered for this cohort of Terena infants suggest that the impact of this package of policies and programs to protect, promote, and support breastfeeding was limited/contained among indigenous communities.

This is the first study to describe practices of breastfeeding among indigenous peoples living in an urban area of Brazil. In addition, we used of accrual method to measure duration of exclusive breastfeeding to minimize inaccuracy on its estimation due to memory bias ^68^ ^,^ ^69^. Thus, in three different time-points during the first year of life, in addition to asking mothers how long they breastfed exclusively, mothers were asked about the age when baby first received any liquids or foods other than breast milk. A limitation of this study is that even though the study enrolled all Terena infants born in the city of Campo Grande within an established period, our findings cannot be directly generalized to other urban indigenous contexts in Brazil or to other indigenous ethnicities living in Campo Grande. Furthermore, the comparisons of prevalence of exclusive breastfeeding duration according to the sociodemographic and health characteristics of the Terena mothers and infants should be made carefully given the small number of observations, which may have made difficult for associations to reach statistical significance.

## Conclusions

The low rates of exclusive breastfeeding duration until the age of three and six months and the high prevalence of bottle use among the Terena infants of Campo Grande contrasted importantly with the rates of exclusive breastfeeding duration and the prevalence of bottle use registered among Brazilian non-indigenous infants. This study reinforces the importance of further studies and initiatives for monitoring the indicators of early life nutrition among Brazilian indigenous peoples, in special those living in urban areas. This is an important gap in the current knowledge of the indigenous health profiles and their determinants in Brazil, with all its implications for their health care.
